# Novel Regulatory Mechanisms of Pathogenicity and Virulence to Combat MDR in *Candida albicans*

**DOI:** 10.1155/2013/240209

**Published:** 2013-09-16

**Authors:** Saif Hameed, Zeeshan Fatima

**Affiliations:** Amity Institute of Biotechnology, Amity University Haryana, Manesar, Gurgaon 122413, India

## Abstract

Continuous deployment of antifungals in treating infections caused by dimorphic opportunistic pathogen *Candida albicans* has led to the emergence of drug resistance resulting in cross-resistance to many unrelated drugs, a phenomenon termed multidrug resistance (MDR). Despite the current understanding of major factors which contribute to MDR mechanisms, there are many lines of evidence suggesting that it is a complex interplay of multiple factors which may be contributed by still unknown mechanisms. Coincidentally with the increased usage of antifungal drugs, the number of reports for antifungal drug resistance has also increased which further highlights the need for understanding novel molecular mechanisms which can be explored to combat MDR, namely, ROS, iron, hypoxia, lipids, morphogenesis, and transcriptional and signaling networks. Considering the worrying evolution of MDR and significance of *C. albicans* being the most prevalent human fungal pathogen, this review summarizes these new regulatory mechanisms which could be exploited to prevent MDR development in *C. albicans* as established from recent studies.

## 1. Introduction

In the last decades, the incidence of fungal infections has increased dramatically due to the rise in the number of immunocompromised patients. The most prevalent fungal pathogen of humans is *Candida albicans* which ranks as the fourth most common cause of hospital acquired infectious disease and is the primary cause of systemic candidiasis, with mortality rates approaching 50% [1]. The dimorphic opportunistic pathogen, *C. albicans*, is normally a commensal organism in humans, but when the host is unable to mount an adequate immune response, as in AIDS, organ transplant, diabetes, or in cancer patients, it results in mucosal, cutaneous, or invasive mycoses [2, 3]. Prolonged usage of antifungals in treating infections caused by *C. albicans* has led to the emergence of azole resistance. This acquired azole resistance in clinical isolates of *C. albicans* mostly results in cross-resistance to many unrelated drugs, a phenomenon termed multidrug resistance (MDR) [4–6]. MDR is a serious complication during treatment of opportunistic fungal infections which poses grave concern given the limited number of clinically useful antifungal drugs available [7, 8]. Fungal species have evolved a multitude of mechanisms to survive exposure to antifungal drugs and some of them include an overexpression or mutations in *ERG11*, encoding the target enzyme of azoles lanosterol 14*α*-demethylase [4, 5, 9, 10], an over expression of the drug efflux pumps encoding genes such as *CaCDR1* and *CaCDR2* belonging to the ABC (ATP-binding cassette) [11–13] and *CaMDR1* belonging to the MFS (major facilitator super family) transporters [14–16].

**Figure 1:**
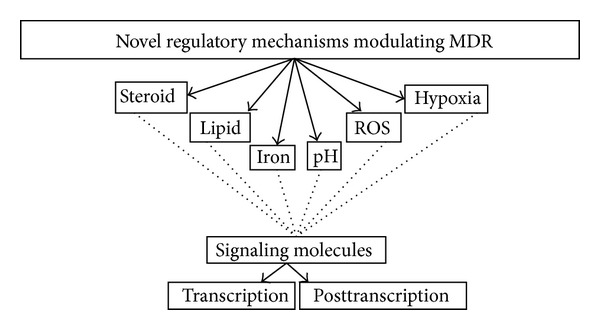
Novel regulatory mechanisms modulating MDR in *Candida albicans*.

Although MDR is a complex manifestation of factors which are reasonably documented, there are reports to suggest that it may involve many unknown mechanisms which are yet to be elucidated. In the recent years, emerging evidence has demonstrated that there do exist such novel mechanisms which can be helpful in controlling MDR efficiently. Improved knowledge of such molecular mechanisms controlling MDR in pathogenic fungi should facilitate the development of novel therapies to combat these intransigent infections. This review further defines the focus on the exacerbated need of understanding such mechanisms (Figure 1) and attempts to highlight research areas that need to be investigated in greater detail.

## 2. ROS 

In eukaryotic cells, mitochondria are common organelles that represent an important source of reactive oxygen species (ROS). ROS are not just a byproduct of mitochondrial disintegration but also a key regulator for yeast apoptosis [17]. As mediators in signal transduction pathways, ROS participate in early and late steps of the regulation of apoptosis. ROS produced by granulocytes or monocytes are known to exert activity against fungi [18]. Furthermore, *C. albicans *possesses a ROS scavenger, superoxide dismutase suggesting that fungi may require a cytoprotective mechanism against not only exogenous ROS but also endogenous ROS [19]. Kobayashi [20] demonstrated the role of ROS in azoles mediated drug sensitivity in *C. albicans,* thereby establishing a strong correlation between ROS and MDR. They showed that ROS production is directly involved in the cytostatic action of miconazole. In this study complete inhibition of miconazole-induced ROS production resulted in the restoration of 50 to 70% of cell viability, suggesting that ROS production is an important event, in addition to drug-induced inhibition of ergosterol synthesis. Similarly Wu [21] deciphered the antifungal action of plagiochin E (PLE) through mitochondrial-dysfunction-induced ROS accumulation in *C. albicans*. Xu [22] showed that endogenous ROS augmentation contributes to the synergistic action of fluconazole (FLC) and berberine against FLC-resistant *C. albicans.* Rosa [23] explored a mechanistic link between the drug sensitivity, gene expression, and pathogenesis phenotypes of *C. albicans*. They conclude that histone acetyltransferase, Rtt109, is particularly important for fungal pathogenicity, suggesting a unique target for therapeutic antifungal compounds. Recently, the fungicidal activity of Gemini-pyridinium salts and shikonin was found to be mediated through ROS generation only [24, 25]. Thus the fact that ROS production could either form the basis of antifungal action of the compound mentioned above or act in synergism to enhance the cytotoxicity of drugs has become a potential therapeutic strategy nowadays. Therefore, ROS signaling pathways need to be further elucidated in fungal pathogens to enhance the potency of such target.

## 3. Iron

Pathogens including *C. albicans* colonize various niches which are iron-limited, and iron, being an indispensable micronutrient, is required both by the host and by the microbial community residing within the host [26–31]. Availability of iron in host cells is tightly regulated, since iron is a transition metal and its ability to donate and accept electrons can indulge in the formation of toxic free radicals, and hence iron plays a key role in providing natural resistance to infections in humans [32]. Interestingly, studies suggest that there could be a correlation between intracellular iron and MDR phenomenon. For instance, role of iron in recurrent vulvovaginal candidiasis (RVVC) showed that this elements is not only important for pathogenic yeast, but also for normal function of host immunity [30]. Kuipers [33–35] showed that lactoferrin, an iron binding glycoprotein, is synergistic with antifungals against different *Candida *species. However, whether iron affects drug susceptibility of *Candida* cells was not demonstrated experimentally until Prasad [36] reported for the first time that availability of iron could have an impact on defense mechanisms of *Candida* against antifungal drugs. Interestingly, it was observed that iron deprivation enhanced drug susceptibility of *Candida* cells resulting in an increase in membrane fluidity, which in turn leads to enhanced passive diffusion of drugs. A link between changes in membrane fluidity and lowered ergosterol levels was established in iron deprived *Candida* cells probably due to downregulation of *ERG11*. However, the intricate relationship between cellular iron, calcineurin signaling, membrane lipid homeostasis, and drug susceptibility of *Candida *cells was first established by Hameed [37]. Even antifungal action of malachite green is mediated via depletion of labile iron pools as one of its mechanisms [38]. The synergism of lactoferrin with fluconazole has been reported to enhance the antifungal activity of fluconazole against *Candida *spp. [39]. Cap2/Hap43 is essential for *C. albicans *growth under iron-deprivation conditions and for virulence in mouse [40]. Moreover, the usage of fungicidal monoclonal antibodies seems to interfere with iron acquisition in *C. albicans *[41]. Many transcription factors governed by iron homeostasis namely, Sfu1, Hap43, Sef1, Cap2, and Aft2, and regulators of iron uptake genes have already been identified; however, sufficient knowledge of signaling pathways is still lacking, and therefore, understanding the role of these transcription factors which are governed by iron availability will better help in studying the relation between iron and MDR. Kaba [42] demonstrated a link between mitogen activated protein kinase HOG1 and iron availability. Dap1 is a heme binding protein that mediates a functional link between iron homeostasis and azole resistance in *C. glabrata* [43]. Since dap1 mutants show enhanced azole susceptibility and deceased ergosterol production, hence, its role in *C. albicans* could also be a possible target. Gene expression profiling of BCR1 transcription factor, known to have a role in biofilm formation, has reported that five out of eight genes have been implicated in iron homeostasis [44]. The influence of cellular iron on drug susceptibilities of *Candida *suggests iron to be yet another novel determinant of MDR which merits a closer look. 

## 4. Hypoxia 

Organisms often encounter sites within the host during infection which have inadequate vascularization and irregular blood flow and thus present hypoxic areas. The transcription factor complex hypoxia inducible factor 1 (*HIF-1*) controls the expression of most genes involved in adaptation to hypoxic conditions and therefore represents an important regulator of MDR development. Hypoxia is known to cause resistance to chemotherapy by the induction of human *MDR1* in growing tumors, via activation of hypoxia inducible factor-1 (*HIF1*) [45–48]. Although till date no homolog of *MDR1* has yet been identified in *C. albicans*, as this organism encounters sites during infection which are hypoxic, it is indeed inevitable to understand the hypoxic responses of this pathogen. The relevance to explore hypoxic responses in *C. albicans* is depicted from a wide range of studies. For instance, transcriptome response of *C. albicans* under hypoxia reveals metabolic adaptation to scarce amount of oxygen availability [49]. Following hypoxic growth in vagina-simulative conditions, part of *C. albicans* proteome that is covalently linked to the cell wall has been determined [50]. Efg1 plays a major role in hypoxic responses of *C. albicans* as suggested by the fact that *efg1* mutants, though inhibit hyphae formation during normoxia, are able to express filaments under microaerobic conditions if grown on or within (embedded) agar at temperatures from 25°C to 35°C [51, 52]. Another study revealed that efg1 mutants are hyperfilamentous as a response to low oxygen [49]. Role of Efg1p in biofilm formation, which is one of the major threat towards antifungal chemotherapy, under hypoxia has been established where Efg1p induces all the major classes of genes required for biofilm formation [53]. Moreover, adaptation to hypoxia forms an integral component of biofilm formation in *C. albicans* [54]. Carvalho [55] demonstrated that aspartyl protease activity could be modulated by oxygen availability in *C. albicans*. Another study revealed that regulation of gene expression in response to hypoxia in *C. albicans* could be signaled via lowered sterol levels and induction of filamentation under hypoxic conditions requires the Ras1- and Cdc35-dependent pathway [56]. Recently, it has been demonstrated that kinase Sch9 integrates both hypoxia and CO_2_ sensing to inhibit yeast to hyphal transition in *C. albicans* [57]. Responses to hypoxic conditions in pathogenic fungi including *C. albicans* have been already extensively reviewed [58, 59]. Certainly further research on the effect of hypoxia on infection by fungal pathogens should be focused. Antifungal compounds that act specifically on adaptive mechanisms of pathogens required for hypoxic adaptation could be promising alternatives to existing strategies.

## 5. Steroids

Steroids have been known to affect cell growth, germination, morphogenesis, and virulence in fungi [60–62]. Some lines of evidence for the presence of steroid binding proteins in yeast, such as estradiol binding protein [63], corticosteroid binding protein [64], and progesterone binding protein [65], have already been documented but their exact function in the steroid response is not known. Many studies have shown that steroids can induce a pleiotropic drug resistance (PDR) state in both pathogenic *C. albicans *and nonpathogenic *Saccharomyces cerevisiae*. Oestrogen regulation and its mechanism have been extensively studied [66, 67] and it is reported that the *CDR1 *promoter region does contain steroid responsive elements (SRE) and a drug response element (DRE). Oestrogen mediated binding of the transcription factor *TAC1 *to the DRE and induction of *CDR1 *expression have been studied [68]. Banerjee [69] for the first time demonstrated that yeast cells, which do not possess steroid receptor cascade, probably perceive steroids as a cellular stress. They examined the genome-wide changes in the gene expression profile following exposure to progesterone. Interestingly, an inverted CCAAT box which in combination with other conserved sequences is attributed towards human *MDR1* responsiveness to cellular stresses is also present in steroid responsive region consensus sequences [70–72]. Banerjee [73] deciphered a more accurate evolutionary significance of the steroid response in yeasts by exposing *S. cerevisiae *and *C. albicans *cells to several doses of progesterone for different time periods. The study revealed the conserved and divergent features of PDR network in yeasts. Recently, antifungal potentials of steroidal quinolones and chalcones, respectively, have been reported and results seems to be quite promising [74, 75]. These studies showed that the steroid response in the absence of any known signaling cascade is a global phenomenon in yeast cells. Yet, in view of the importance of steroids in the physiology of both pathogenic and nonpathogenic yeasts, it would be interesting to examine the steroid-dependent regulatory cascade in these organisms and thereby decipher a new mechanism to combat MDR.

## 6. Morphogenesis 

In response to various environmental stimuli, *C. albicans *is able to switch from the unicellular yeast form into either of the two distinct filamentous forms, that is, cells with pseudohyphae or true hyphae. This ability to switch is considered as an important virulence trait which is also coregulated with other virulence factors that are associated with cellular morphology [76, 77]. The morphological form of *C*.* albicans* is directly related to environmental conditions and these cues trigger separate signal transduction pathways which regulate common targets required to initiate hyphal growth [51, 76, 77]. The transcription factor Efg1p is a well known regulator of morphogenesis of *C. albicans* since it induces the yeast-to-hyphal transition and also regulates phenotypic switching and chlamydospore formation of this pathogen [52]. 

Lo [78] established that Efg1p is involved in drug resistance by regulating the expression of *ERG3* gene of ergosterol biosynthetic pathway. Ergosterol is an important target for many antifungal drugs particularly on the plasma membrane. Considering the significance of Efg1p regulator in morphogenesis, Prasad [79] have evaluated if disruption in morphogenic signaling cascade would also affect MDR status of *C. albicans* cells. The study showed that null mutant of the morphogenic regulator *EFG1* displayed enhanced drug sensitivity of *C. albicans* cells by a mechanism that is not dependent on the drug efflux pumps. This study establishes a convergence of *EFG1* and *MDR* pathways and thus proposes an additional new role for this important morphogenic regulator of *C. albicans.* The role of a newly discovered regulator of hyphae formation, Rca1, in drug susceptibility of *C. albicans* was established recently [80]. Certainly more extensive analyses are required to elucidate the commonality between *EFG1 *and MDR signaling cascades to find newer targets for antifungal chemotherapy.

## 7. pH 

The ability of microorganisms to sense and adapt to changes in the environment is essential for their survival. One environmental factor that microorganisms must respond to is extracellular pH. Environmental pH has dramatic effects on the cell, particularly at the plasma membrane, including effects on protein activity, maintenance of the proton gradient, and nutrient availability. Furthermore, in the opportunistic fungal pathogen *C. albicans*, environmental pH serves as one potent signal for morphological differentiation, although serum N-acetyl glucosamine and agar-embedding represent other several potent signals [2, 3]. Most chemotherapeutic drugs in use today are hydrophobic small, molecules that are also typically either weakly basic, weakly acidic, or charged. Thus, changes in the electrochemical parameters of microorganism's cell membranes have important effects on their transmembranous diffusion and cellular retention [81]. Changes in these parameters can also modulate the function of immunological agents and affect the signal transduction. The *RIM101* pathway that has been identified in *C. albicans* governs pH responses, dimorphism, and pathogenesis [82]. The *RIM101* pathway and pH responses, in general, play an intimate role in pathogenesis beyond simply allowing the organism to grow [83]. Apart from the major *RIM101* pathway, some other pathways have also been identified in *C. albicans, *which act in parallel. For instance a novel *RIM101 *independent pH pathway was proposed which was mediated by *PHR2 *[83]. Phr1p and Phr2p are differentially expressed cell wall proteins in response to environmental pH. Phr1p is expressed under alkaline conditions and Phr2p is expressed under acidic conditions, and importantly both are required for the pathogenesis of the organism for causing systemic candidiasis (pH 7.4) and vaginal candidiasis (pH 4) [84]. Another signaling pathway that was identified for adaptation to neutral/alkaline pH is calcineurin signaling [85]. Calcineurin is a highly conserved calcium-dependent serine/threonine-specific protein phosphatase that mediates various stress responses inside the cell including conferring tolerance to alkaline pH in *C. albicans *[86]. Furthermore, Hameed [37] provided the first evidence to establish relationship between cellular iron, calcineurin signaling, membrane lipid homeostasis, and drug susceptibility of *Candida *cells. They showed that iron deficiency leads to downregulation of calcineurin signaling pathway leading to abrogated sensitivity at alkaline pH, salinity, and membrane stress. In general the ability of pathogenic fungi to adapt to host pH is critical for survival and disease progression. This highlights the importance of continuing studies of these fundamental pH response pathways in pathogenic fungi in order to understand how these pathogens are adapted to the mammalian host and potentially identify new approaches for preventing or treating infections.

## 8. Lipid 

Recent lines of evidence have provided a comprehensive amount of data regarding the role of lipids and many other lipid derivatives in establishment of various infectious diseases. MDR in yeast is closely linked to the status of membrane lipids. It has been already established that the associated changes in membrane lipid composition (phospholipid and ergosterol), its order (fluidity), and asymmetry could be important determinants in the drug susceptibilities of *Candida* cells [87]. Similarly, changes in membrane lipid composition between sensitive and resistant could also influence the action of antifungal drugs like azoles and thereby can form one of the factors responsible for drug resistance [12]. Most strikingly it has been observed that clinical as well as adapted azole-resistant isolates of *C*. *albicans *exhibit altered membrane phospholipid and sterol compositions [88]. Mukhopadhyay [89] demonstrated that there is an interaction between membrane ergosterol and sphingolipids, and a reduction in the content of either of these two components results in a disruption of this interaction, which has deleterious effects on the drug susceptibilities of *C*. *albicans *cells. Thus the fact that lipid could also play an important role in drug susceptibilities is becoming apparent from a wide range of recent studies. The importance of lipid signaling molecules in the development and pathogenicity of clinically important fungi has been highlighted [90]. In *C. albicans*, exposure to the oxylipin farnesol causes the regulation of specific genes involved in hyphal development, drug resistance, and iron acquisition. Farnesol increases resistance to oxidative stress in *C. albicans*. Through technologies such as lipidomics, a wider vision has been obtained to know about the diversity of lipid molecule which is not only limited to cellular functions but also to pathogenesis of diseases. Nowadays, lipidome-wide quantification of individual molecular lipid species (molecules with defined chemical structure) by absolute quantification provided a new approach to relate lipidomics and functional genomics studies [91]. Hameed [37] demonstrated the role of lipid homeostasis in iron mediated drug susceptibility of *C. albicans *where iron deprivation leads to enhanced drug susceptibility due to lowered ergosterol levels and increases membrane fluidity. Similarly the antifungal effect of curcumin has also been shown to be influenced by membrane lipid composition depicting marked changes in phosphoglyceride (PGL) species and also ergosterol depletion [92]. Recently, a high throughput lipid profiling has revealed some differences in lipid composition between azole sensitive and azole-resistant isolates highlighting fluctuations in phosphatidyl serine, mannosylinositolphosphorylceramides, and sterol esters levels indicating their compensatory role in maintaining lipid homeostasis among most of the resistant *Candida* isolates [93]. Cross-talk between mitochondrial lipid homeostasis, cell wall integrity, and azole tolerance has been revealed showing significant changes in several lipid classes, particularly in plasma membrane microdomain-specific lipids such as mannosylinositolphosphorylceramides and ergosterol, and in a mitochondrial-specific phosphoglyceride, phosphatidyl glycerol [94]. The overall cellular lipid homeostasis is a critical factor in the observed FLC resistance [95]. Although the transcriptome, proteome, and interactome of several eukaryotic model organisms have been described in detail, lipidomes still remain relatively uncharacterized and will also improve our understanding of the molecular architecture of membrane domains and cellular organelles. Hence, the lipid associated changes of pathogenic fungi induced in response to infection might help in better understanding of the role of lipids in pathogenesis of *C. albicans* and thereby development of better therapeutic strategies.

## 9. Signaling 

A focal point among the fungal pathogens is that signaling molecules have a key role in mediating cellular stress responses. Signal transduction pathways are crucial mechanisms that allow cells to sense and respond to diverse environmental cues. Exploiting these stress responses through blocking of signaling pathways may provide the foundation for new combination therapies to enhance the efficacy of our limited resources of clinically useful antifungal drugs. Jain [96] have examined the relationship between azole susceptibility and the cyclic AMP (cAMP) protein kinase A (PKA) signaling pathway. Likewise, a key regulator of cell signaling in all eukaryotes, calcineurin, provides a perfect example of the role of signaling molecules in mediating crucial responses to antifungal drugs [97]. Alonso-Monge [98] describe that MAPK cascades control most of the virulence factors characterized in *C. albicans*: cell morphology, superficial antigen (cell wall biogenesis), and response to oxidative and nitrosative stresses. These cascades allow opportunistic pathogens to recognize changes in their environment and take advantage of an impaired immunological system to cause infection. Recent studies reveal that Hsp90, a component of a chaperone complex induced by heat stress, governs drug resistance in fungi [99, 100]. Bastidas [101] showed the significance of signaling cascades in pathogenic fungi. They not only depicted the well known calcineurin pathway and that Hsp90 is an important antifungal target but also described the TOR signaling pathway in context with antifungal drug resistance. Thakur [102] showed mechanistically similar regulation of MDR like in vertebrates by the PXR nuclear receptor, revealing an unexpected functional analogy of fungal and metazoan regulators of MDR. Robbins [103] establish a novel role of nutrient signaling in azole resistance. They revealed that compromising the function of Tor kinase, a global regulator of growth and metabolism, could be an efficient strategy to control drug resistance. Another key cellular stress response pathway having implications in basal tolerance to azoles is the protein kinase C (PKC) mediated cell wall integrity pathway (CWI). Lafayette [104] established a novel role of CWI and calcineurin signaling pathway in *C. albicans,* while de Dios [105] revealed the relevance of MAPK signaling cascades in fungal virulence. The emerging roles of GlcNAc as an activator and mediator of cellular signaling in *C. albicans* have also been reviewed [106]. Recently, even the significance of metabolic pathways as therapeutic targets and how their disruption can have both physiological and regulatory consequences have also been demonstrated [107]. The diterpene acid, phorbasin H, affects the activity of the cAMP-Efg1 pathway leading to an alteration of *C. albicans* morphology as was demonstrated recently [108]. Likewise antidimorphism activity of catechin was depicted by interfering with Cek1 phosphorylation and cAMP synthesis [109]. Kaba [42], demonstrated the involvement of MAPK HOG1 pathway with iron availability, and as already discussed in the previous sections, iron plays a vital role if one considers drug susceptibility in *C. albicans*. A new study showed for the first time that even lower eukaryote like *C. albicans* possesses a two-component response regulator protein [110]. Thus a deeper knowledge of the mechanism and regulation of various signaling cascades could help in the control of candidiasis as well as in the development of effective therapeutics against these severe infections.

## 10. Transcription Factors

Transcriptional regulation can be of significant importance in the development of antifungal resistance. It is important to understand the regulatory network controlling drug resistance in fungal pathogens. Different strategies have been adopted for isolation of regulators of multidrug transporters in *C. albicans*. One such approach is through the analysis of cis-acting elements in genes encoding multidrug efflux transporters. For instance the basal expression element (BEE) responsible for basal expression, the drug-responsive element (DRE) required for the response to drugs such as fluphenazine and estradiol, two steroid-responsive element (SRE) involved in the response to steroid hormones and the negative regulatory element (NRE), have been already deciphered [66, 67, 111]. One of the major breakthroughs was the discovery of *TAC1* regulator whose deletion abrogates the CDR1/CDR2 expression in *C. albicans* clinical isolates resistant to azoles, thus demonstrating that *TAC1* was a major mediator of azole resistance due to the upregulation of the ABC transporter [68]. Apart from it other potential regulators of *CDR1* have also been reported which were identified through functional complementation in *S. cerevisiae*. For instance functional homologue of PDR1/PDR3 in *S. cerevisiae,* namely, fluconazole resistance 1 (*FCR1*), was reported in *C. albicans*. Deletion of *FCR1* in *C. albicans* resulted in reduced susceptibility to FLC [112]. Similarly, *NDT80* inactivation in *C. albicans,* a gene similar to the *S. cerevisiae NDT80* gene, resulted in a decreased basal *CDR1* expression and a decreased CDR1 inducibility in the presence of drugs [113]. Genome-wide transcription profiling was employed for the identification of an *MDR1* regulator or of factors binding to the MDR1 promoter. By comparing the transcriptional profiles of three different *C. albicans* clinical isolates over expressing *MDR1* with azole-susceptible parents, one of the commonly upregulated genes in the three isolates was orf19.7372 which was subsequently known as *MRR1* [114]. Another study reported that regulator of efflux pump 1 (*REP1*) acts as a negative regulator of *MDR1* which belongs to the transcription factor family including *NDT80* [115]. Similarly, *C. albicans* gene (*UPC2*) with homology to both *S. cerevisiae* genes has been identified and characterized [116]. Deletion of *UPC2* in *C. albicans* abrogates *ERG11* upregulation in response to azole drugs. A recent study has demonstrated the role of the mediator complex in the transcriptional response of multidrug transporter genes in *S. cerevisiae *and *C. glabrata* [102]; however, in *C. albicans*, the binding to the transcriptional activator of drug resistance genes (*TAC1, MRR1,* and *UPC2*) still remains hypothetical.

Gain-of-function (GOF) mutations in alleles of the transcription factors (*TAC1, MRR1, CgPDR1,* and *UPC2*) from azole-resistant isolates cause constitutive high expression of their drug resistance gene targets and thus azole resistance when expressed in an azole-susceptible background [68, 114, 117–122]. Nowadays, genome-wide analysis is an important tool to yield regulons of selected transcription factors for the elucidation of transcriptional regulatory network of drug resistance. Moreover, systematic deletion of transcription factor genes could also be an alternative approach to reveal transcriptional circuits responsible for drug resistance. Genome-wide genetic screens for the identification of additional targets involved in drug resistance or participating in the response of fungal pathogens to drug exposure will likely result in elucidating regulatory mechanisms. Recently, it has been uncovered that a network comprises 800 target genes and a tightly knit transcriptional regulatory circuit indicating that many aspects of commensalism and pathogenicity are crosslinked [123]. Dhamgaye [124] deciphered through RNA sequencing some novel genes including the transcription factor *CZF1* which are responsible for drug resistance.

Although the transcriptional regulation is considered to be the major step in MDR regulation, reports do exist nowadays revealing the significance of posttranscriptional regulation as well. Manoharlal [125] demonstrated that *CDR1* mRNA half-life was increased in azole-resistant as compared to azole sensitive isolates. Moreover, Manoharlal [126] further dissected the molecular basis of the above observed increased mRNA stability where it was demonstrated that loss of heterozygosity at the *PAP1* locus is linked to hyperadenylation and subsequent mRNA stability of *CDR1* transcripts in azole resistant isolates. Recently, posttranscriptional regulation of transcription factor Sef1 has been demonstrated in controlling virulence of *C. albicans* [127]. Thus despite poor knowledge about the posttranscriptional regulation of MDR in *C. albicans*, certainly, this aspect needs to be further elucidated to gain deeper insights.

## 11. Concluding Remarks 

Today, the rapidly evolving issue of MDR demands the obvious need for dissecting completely new regulatory mechanisms that could be targeted to control MDR. Novel mechanisms described earlier for pathogenic fungi clearly hold promise that can facilitate the development of better antifungal strategies to efficiently control the human fungal diseases. Elucidating these mechanisms may provide new foundations for antifungal chemotherapy and can present an exciting challenge for the future investigation.
